# Otosyphilis: A Rare Cause of Reversible Hearing Loss in a Teenage Male

**DOI:** 10.7759/cureus.23468

**Published:** 2022-03-24

**Authors:** Shan He, Anna H Messner, Gayatri Mirani

**Affiliations:** 1 Anesthesiology, Texas Children’s Hospital/Baylor College of Medicine, Houston, USA; 2 Otolaryngology-Head and Neck Surgery, Texas Children’s Hospital/Baylor College of Medicine, Houston, USA; 3 Immunology, Allergy, and Retrovirology, Texas Children’s Hospital/Baylor College of Medicine, Houston, USA

**Keywords:** sexually transmitted diseases, aids, hiv, hearing loss, otosyphilis, neurosyphilis, syphilis

## Abstract

A high index of suspicion and a thorough neurotologic examination at the onset of presentation are imperative to generate the diagnosis of otosyphilis. Complete audiologic recovery is rare but possible in approximately 20%-25% of patients after appropriate treatment. We present a case of reversible hearing loss secondary to otosyphilis in a teenage male patient with a new diagnosis of human immunodeficiency virus (HIV). Audiology findings were consistent with mixed hearing loss. Lumbar puncture results were consistent with neurosyphilis. Prompt treatment with a 14-day course of intravenous penicillin led to the complete recovery of hearing. In this case report, the pathophysiology, symptomology, and management of otosyphilis are discussed.

## Introduction

Cases of syphilis continue to be prevalent worldwide; the incidence of syphilis is also associated with concurrent human immunodeficiency virus (HIV) infections [1]. In adolescent patients with the presentation of a classic syphilitic rash, coupled with neurologic or ophthalmic involvement such as hearing loss or cranial nerve deficits, disseminated syphilis should always be considered as part of the differential diagnosis. Otosyphilis is a rare presentation involving sudden and fluctuating sensorineural hearing loss. The diagnosis of otosyphilis is often delayed or simply missed by physicians due to its ability to mimic a wide spectrum of audiovestibular diseases [2]. At-risk patients should undergo thorough neurologic, otologic, and ophthalmic examinations given that otosyphilis is one of the rare reversible causes of sensorineural hearing loss.

## Case presentation

A 16-year-old male patient presented with a complaint of rash. His past medical history was significant for gonorrhea and chlamydia infections one year prior. Three months prior to presentation, he noticed dry patches on his extremities. Two weeks later, a maculopapular rash appeared on his groin, axilla, and thorax. Six weeks prior to presentation, a brown discoloration formed on his palms and soles. An additional review of systems revealed a 20-lb weight loss over the same time span. His social history was significant for unprotected sexual intercourse with three male partners since the age of 14.

On the initial examination, the patient appeared thin with a body mass index of 18.4 kg/m². During the neurologic examination, no deficits were noted on the cranial nerve and motor examinations. He demonstrated normal gait, coordination, and balance but required frequent repetition of verbal questions, raising clinical concern for hearing loss. On the otologic examination, he was found to have bilateral serous middle ear effusions. The oral cavity examination was significant for shallow ulcerations on his palate and scattered white patchy lesions on the mucosa.

**Figure 1:**
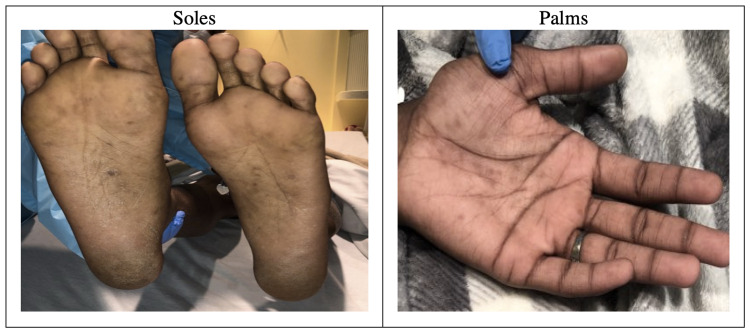
Dermatologic Findings on Physical Examination

Significant dermatologic findings are shown in Figure 1. The examination demonstrated diffuse dry skin with mild flaking on bilateral lower extremities and upper arms. Clusters of brown papular and verruciform rash were present on his upper torso, abdomen, genitals, and buttocks. Flat brown patches were present on the patient’s palms and soles. There were enlarged non-tender lymph nodes in the neck and bilateral inguinal areas that were soft and mobile, ranging between 2 and 3 cm. The genitourinary examination was significant for perianal warts with marked tenderness.

The fourth-generation human immunodeficiency virus (HIV) test was positive. The patient’s initial HIV RNA was 139,013 copies/mL with a CD4 count of 280 cells/μL and a CD4% of 14%. The patient was immediately started on antiretroviral therapy with bictegravir, emtricitabine, and tenofovir alafenamide, a fixed-dose combination pill. A *Treponema pallidum* (TP) antibody test was positive with a rapid plasma reagin (RPR) titer of 1:512. The patient was given his first dose of intramuscular benzathine penicillin G 2.4 million units for the treatment of secondary syphilis.

**Figure 2:**
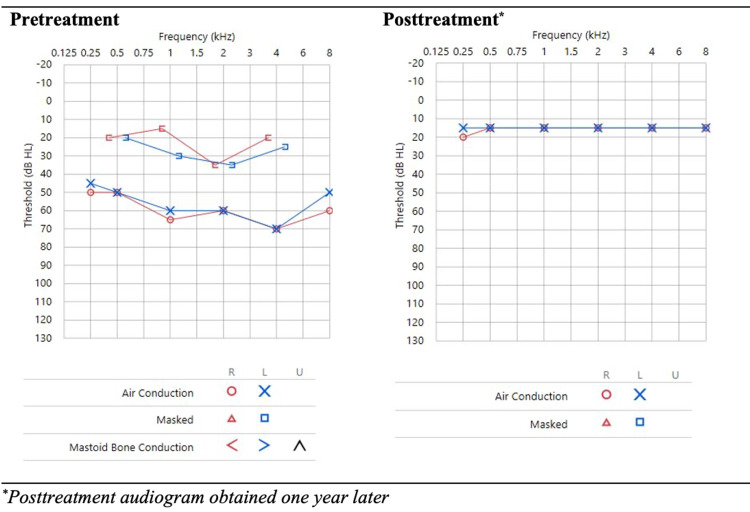
Pretreatment and Posttreatment Audiograms

**Figure 3:**
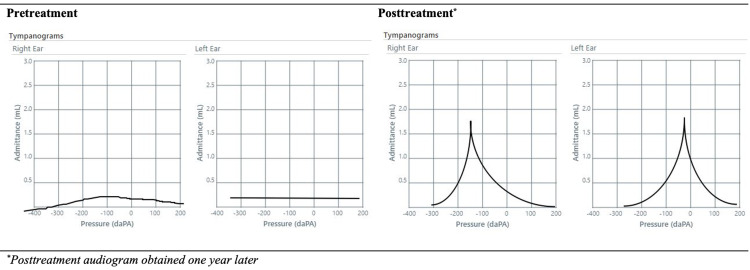
Pretreatment and Posttreatment Tympanograms

Given the patient’s subjective hearing loss, an otolaryngology consultation and an audiology diagnostic evaluation were completed. The study revealed a moderate mixed (conductive and sensorineural) hearing loss. Acoustic immittance measures were consistent with a middle ear disorder (Figure 2). The ear tympanograms were abnormally flat bilaterally (Figure 3). While the serous effusion seen on the physical examination explained the conductive portion of the hearing loss, it did not explain the component of sensorineural hearing loss, which strengthened the case for the diagnosis of otosyphilis. The middle ear effusions were suspected secondary to eustachian tube dysfunction secondary to nasopharyngeal inflammation or a recent episode of acute otitis media, unrelated to his syphilis diagnosis.

A lumbar puncture was performed. His cerebrospinal fluid (CSF) results were concerning due to an abnormal number of lymphocytes and a slightly elevated protein level. While the CSF Venereal Disease Research Laboratory (VDRL) test was nonreactive, his CSF fluorescent treponemal antibody test result returned positive, confirming a diagnosis of neurosyphilis (Table 1). Ophthalmology evaluation did not reveal any ocular abnormality.

**Table 1:** CSF Results

	Pretreatment	Posttreatment	Reference ranges
Cell count	16 white blood cells (95% lymphocytes, 5% macrophages), 0 red blood cell	1 white blood cell, 19 red blood cells	0-5/CU
Glucose	44 mg/dL	62 mg/dL	50%-70% of serum glucose
Protein	48 mg/dL	27 mg/dL	15-45 mg/dL
VDRL test	Nonreactive	Nonreactive	Nonreactive
*Treponema pallidum* antibody	Reactive	Minimally reactive	Nonreactive

The patient remained in the hospital and completed 14 days of treatment for neurosyphilis and otosyphilis with intravenous penicillin G potassium 4 million units every four hours. At subsequent outpatient visits, he demonstrated longitudinal clinical improvement. Repeat lumbar puncture at 10 months demonstrated normalization of CSF values (Table 1). Ten months after initiating antiretroviral therapy, his viral load decreased from a baseline HIV RNA of 139,013 to 42 copies/mL, and his CD4 count improved to 494 cells/μL with a CD4% of 24%. RPR titer was reduced to 1:4. Repeat audiology evaluation a year after initial presentation showed normal hearing (Figure 2) and tympanograms (Figure 3), and the patient reported clinical resolution of hearing loss.

## Discussion

Syphilis is a sexually transmitted infection caused by the spirochete* Treponema pallidum*. The presentation of syphilis infections can be categorized into four stages as presented in Table 2 [1-3].

**Table 2:** Stages of Syphilis

Stage	Presentation
Primary syphilis	Localized infection, painless chancre at the inoculation site
Secondary syphilis	Systemic infection, maculopapular skin rash characteristically involving the palms and soles, lymphadenopathy
Tertiary syphilis	Wide range of neurologic, cardiac, and skin findings including gummatous lesions
Latent syphilis	Serologic signs of infection without clinical symptoms

Otosyphilis is a rare manifestation of systemic *Treponema pallidum* bacterial infection, with the most common clinical symptoms including sensorineural hearing loss, tinnitus, and vertigo [4]. Bilateral hearing loss typically occurs acutely with rapid progression, accompanied by vestibulocochlear symptoms [5]. The presentation can also vary between individuals, spanning unilateral and bilateral involvement, asymmetric hearing loss, rapidly progressive to fluctuating hearing loss, spontaneous or provoked disequilibrium, and generalized unsteadiness [6,7]. Audiometric studies often involve the low- and high-frequency ranges [5,8] while sparing middle frequencies [9].

Otosyphilis and neurosyphilis can be diagnosed in all phases of syphilitic infections [4,10].* Treponema pallidum* spirochetes can infect the central nervous system, causing hearing loss by syphilitic inflammation of the perilymphatic space, degeneration of the inner ear structures and temporal bone, and impairment of the eighth cranial nerve [4,9,11-14]. Thus, all patients with a confirmed diagnosis of disseminated syphilis should undergo a thorough ocular, otologic, and neurologic examination [13].

Patients with clinical suspicion for otosyphilis first need to have a positive serology for active treponemal infection. The serologic testing of syphilis involves two steps: the initial nontreponemal test such as RPR or VDRL and the secondary confirmatory treponemal test including *Treponema pallidum* particle agglutination (TP-PA) or fluorescent treponemal antibody absorption (FTA-ABS) [13]. Oftentimes, a lumbar puncture is performed to collect CSF analysis to rule out neurosyphilis. CSF abnormalities typically involve elevated proteins with mononuclear pleocytosis [10,13,15], which can be confounding in a patient concurrently infected with HIV and syphilis, as the CSF findings are similar in individuals infected with HIV [1]. CSF analysis is rarely positive for nontreponemal studies such as VDRL [4], but sensitivity improves significantly with CSF treponemal tests such as FTS-ABS and TP-PA [13].

The diagnosis of otosyphilis is inherently challenging as it requires a high index of clinical suspicion even in the context of confirmatory serology. There does not exist a practical way to perform a treponemal test on the perilymphatic fluid nor a histologic examination of the temporal bone [4,12,16]. Diagnosis is multistep, typically requiring a combination of compatible neurologic, cochlear, and vestibular symptoms, a positive serologic test, CSF abnormalities, and/or audiologic studies [4,6,13]. Otosyphilis is also a diagnosis of exclusion, requiring that hearing loss is not caused by another discernable organic inner ear pathology [4,6,11,15].

The accurate and timely diagnosis of otosyphilis in patients concurrently infected with HIV is challenging. Severe impairment in cell-mediated immunity levied by the virus can facilitate the acceleration of the systemic spread of syphilis, at the same time creating opportunities for other microbiotic invaders [5]. For example, aseptic meningitis, *Cytomegalovirus*, and hepatitis B opportunistic infections can all lead to hearing loss [5]. Various intracranial processes leading to cranial eighth nerve neuropathy would also mimic audiovestibular alterations and hearing loss similar to otosyphilis.

Otosyphilis is typically treated as symptomatic neurosyphilis with intravenous penicillin regardless of CSF findings [4,6,9]. Intramuscular penicillin typically fails to obtain appropriate treponemicidal levels in the cerebrospinal and perilymphatic space [8,9,12,16]. In addition, corticosteroids are sometimes administered to improve the chances of remission of hearing loss by reducing inner ear inflammation [6,9]; however, corticosteroids are typically avoided in HIV-infected individuals to avoid further suppression of cell-mediated immunity [8]. Unfortunately, definitive conclusions on treatment outcomes are difficult given the low incidence of this rare disease [7].

The prompt diagnosis of otosyphilis is critical as early treatment can arrest the progression of audiologic disease [12,17]. Treatment may lead to improvement of hearing function and even complete restoration of hearing abilities [7,10,11] given that syphilis is one of the rare reversible causes of sensorineural hearing loss [12]. Meaningful recovery is more likely to happen with a shorter duration of symptoms [11]. Older patients with chronic, persistent vestibulocochlear and audiologic disturbances are less likely to recover [2,16]. Hearing loss is less likely to improve compared to vestibular symptoms [15], with the literature observing an 80% improvement in tinnitus and vertigo compared to 20%-25% of patients with hearing improvement after appropriate antibiotic therapy [8,12].

## Conclusions

Syphilis, the great mimicker, should always be considered as part of the differential diagnosis for adolescent patients presenting with a rash in both routine and emergent evaluations. Otosyphilis, a rare symptom of syphilis, is often a diagnostic challenge requiring a high index of suspicion from the outset of presentation. This case report is illustrative of the varied manifestations of syphilis in an adolescent male who also had a new diagnosis of HIV infection. A complete neurologic examination, audiometry evaluations, and input from otolaryngology specialists led our team to successfully make a prompt diagnosis. After instituting appropriate and timely antibiotic treatment with intravenous penicillin for 14 days, he had complete audiologic recovery of his hearing loss, which is a rare and appreciated outcome. A case of behaviorally acquired otosyphilis in this age group has not been reported to our knowledge, and data on treatment continues to be sparse.
